# MicroRNA-383-5p Regulates Oxidative Stress in Mice with Acute Myocardial Infarction through the AMPK Signaling Pathway via PFKM

**DOI:** 10.1155/2021/8587535

**Published:** 2021-12-07

**Authors:** Linlin Gao, Zhongbao Ruan, Gecai Chen

**Affiliations:** Department of Cardiovascular Medicine, Taizhou People's Hospital, Taizhou, China

## Abstract

**Objective:**

The purpose of this study is to explore the regulating role of microRNA-383-5p (miR-383-5p) in oxidative stress after acute myocardial infarction (AMI) through AMPK pathway *via* phosphofructokinase muscle-type (PFKM).

**Methods:**

We established the AMI model, and the model mice were injected with miR-383-5p agomir to study the effect of miR-383-5p in AMPK signaling pathways. The target gene for miR-383-5p was reported to be PFKM, so we hypothesized that overexpression of miR-383-5p inhibits activation of the AMPK signaling pathway.

**Results:**

In this research, we found that overexpression of miR-383-5p decreases myocardial oxidative stress, myocardial apoptosis, the expression level of PFKM malondialdehyde (MDA), and reactive oxygen species (ROS) in the myocardial tissues after AMI, and finally, AMI-induced cardiac systolic and diastolic function could be improved.

**Conclusion:**

This study demonstrated that miR-383-5p could reduce the oxidative stress after AMI through AMPK signaling pathway by targeting PFKM.

## 1. Introduction

AMI is the main cause of persistent increases in human morbidity and mortality due to obstruction of coronary blood flow [1]. Although cTnI and cTnT are the gold standard for evaluation of myocardial ischemia, we need the search for new biomarkers. AMI can lead to the formation of scar and the remodeling of left ventricular, including cardiac dysfunction and cardiomyocyte hypertrophy and fibrosis [2]. Therefore, it causes a decrease in cardiac output and ultimately leads to heart failure (HF) [3].

MicroRNAs (miRNAs) are a small noncoding RNAs composed of unequal nucleotides that regulate gene expression by binding to a fully complementary or partially complementary target mRNAs at the posttranscriptional level [4]. miRNAs are involved in gene expression during cell differentiation, inflammation, stress response, and proliferation [5, 6]. Emerging evidences show that miRNAs are involved in the pathogenesis of cardiac diseases [7]. Therefore, it may be possible that miRNAs have therapeutic effects [8]. In addition, some researches have shown that upregulation of miR-383-5p can inhibit cell proliferation [9, 10]. According to investigation, miR-383-5p could regulate cell viability through Wnt/*β*-catenin pathway [11]. It is well known that PFKM mutation is one of the most common lesions in breast cancer [12]. Besides, a previous study has discussed that oxidative stress in cardiomyocytes after AMI can cause cell injury through activated protein kinase (AMPK) signaling pathway [13]. And this research is aimed at investigating the effect of miR-383-5P targeting PFKM *via* AMPK pathway on oxidative stress in AMI.

## 2. Materials and Methods

### 2.1. Prediction of the Target Gene of miR-383-5p Sample Collection

The target gene of miR-383-5p was predicted using the miRDB, PicTar, and TargetScan databases. Then, a correlation analysis was performed on these target genes at the intersection using a String database. We searched for targets with known expression in cardiovascular tissues specifically.

### 2.2. Experimental Animal

In this study, male C57BL/6 mice, the age was six to eight weeks, and weighing 22 ± 5 g were included to be raised by the specific pathogen free (SPF) animal center. The average temperature in the animal center is 20°C, the relative humidity is 50-70%, and the light maintenance day and night cycle is 12 h. The whole process is pellet feeding, free feeding, drinking water, and single cage feeding. The cage was cleaned daily, and the cleaning pad was replaced to keep the cage clean. Mice underwent coronary artery ligation, and we use electrocardiogram measurements for the production of myocardial infarction (MI) [14]. Only mice with significant ST-elevation in the ECG were included in the research. Mice were randomly divided into 4 groups: (1) sham group, (2) AMI+PBS group (AMI mice injected with PBS *via* tail vein), (3) AMI+miR-383-5p agomir group (AMI mice with miR-383-5p agomir injected *via* tail vein), and (4) AMI+miR-383-5p agomir NC group (AMI mice with miR-383-5p agomir NC injected *via* tail vein). Seven days after AMI, echocardiography measurements were performed. This study was approved by the Ethics Committee of Taizhou People's Hospital Animal Center (18-CN-23TZ-EC031).

### 2.3. Echocardiography

Seven days after the AMI model was established, echocardiography was performed using ultrasound system to detect left ventricular end-diastolic volume (LVEDD), left ventricular end-systolic volume (LVESD), and left ventricular end-diastolic volume (LVEDV) and left ventricular end-systolic volume (LVESV). In this study, we evaluated cardiac function by calculating EF and FS.

### 2.4. Culture of H9C2 Cells

H9C2 cells were cultivated with Dulbecco's Modified Eagle Medium (DMEM) culture solution (Gibco, Rockville, MD, USA), which contained 10% fetal bovine serum (FBS) (Gibco, Rockville, MD, USA) and penicillin/streptomycin, and the H9C2 cells cultivate in a culture dish (37°C, 5% CO_2_); we changed the medium every other day. The concentration of 2000 *μ*M H_2_O_2_ was used for the construction of H9C2 cell injury model in vitro [15].

### 2.5. Dual Luciferase Reporter Gene Assay

We analyzed the target gene of miR-383-5p by biological prediction website. To find whether PFKM was a target gene for miR-383-5p, then in this research, we did a luciferase reporter assay for verification. Luc-PFKM-wt or Luc-PFKM-mut was cotransfected into cells with miR-383-5p mimic or its control. Luciferase activity was measured by transfection of H9C2 cardiomyocytes by dual luciferase assay system (Promega, Madison, WI, USA).

### 2.6. MDA Detection

The mouse serum was taken and centrifuged at 3000r for 10 minutes. After taking the supernatant, the serum MDA content was measured using a commercial kit (Jianglai, Shanghai, China).

### 2.7. Flow Cytometry

Detect intracellular ROS levels by flow cytometry. The mice were anesthetized by intraperitoneal injection and sacrificed. The myocardial tissues were separated and placed in 1 mL of ice PBS (Syme fisher technology, Boca Raton, FL, USA) liquid and cut. The cells were filtered through a 200-400 mesh screen and adjust the cell concentration. The prepared 1 mL single-cell suspension was incubated with 5 *μ*l of 2′,7′-dichlorofluorescein diacetate (DCF-DA), and the supernatant was removed by centrifugation and then incubated with 10% FBS. After centrifugation at 4°C, myocardial tissue suspension was prepared. The average fluorescence intensity of the intracellular marker fluorescent probe was measured by flow cytometry (BD FACSC alibur type, Becton-Dickinson (BD), Franklin Lakes, NJ, USA). In the same way, H9C2 cells were treated with H_2_O_2_ in 6-well plate for 4 h. Cells were collected and washed with PBS twice and then resuspended with binding buffer. Annexin V-FITC and propidium iodide (BB-4101-2; BestBio Science, Shanghai, China) staining solution were added. The apoptotic cells were detected by flow cytometry (BD FACSC alibur type, Becton-Dickinson (BD), Franklin Lakes, NJ, USA).

### 2.8. Real-Time qPCR

Myocardial tissue RNA was extracted using TRIzol (Thermo Fisher Scientific, Waltham, MA, USA). RNA pretreated with diethylpyrocarbonate (DEPC) (Beyotime, Shanghai, China) was dissolved in ultrapure water. The RNA concentration of the nanodroplets was measured, and the absorbance at 260 nm and 280 nm was measured. If the A260/A280 was between 1.8 and 2.1, the RNA quality was considered standard and can be used in subsequent experiments. We first synthesize complementary deoxyribose nucleic acid (cDNA) using a reverse transcription kit and then perform PCR amplification. mRNA quantitative analysis was achieved using Prism 7300 Sequence Detection System, and 25 *μ*L reaction system was used including SYBR Green (12.5 *μ*L, Thermo Fisher Scientific, Waltham, MA, USA), 10 *Μ*m of primers (0.5 mL each from the stock, Thermo Fisher Scientific, Waltham, MA, USA), 10.5 *μ*L of water, and 0.5 *μ*L of template. The data was analyzed by the SDS software, and the results were then output to EXCEL for further analysis. Glyceraldehyde 3-phosphate dehydrogenase (GAPDH) serves as an internal reference for other mRNA and U6 as an internal reference for miR-383-5p. The comparison threshold period (Ct) method, that is, the 2^-*ΔΔ*Ct^ method was used to calculate the folding magnification. All the primers are listed in Table 1.

### 2.9. Western Blotting Technology

Four groups of mouse myocardial tissue were taken in an eppendorf (EP) tube, and an appropriate amount of precooled cell lysis buffer was placed. The supernatant was taken by centrifugation at 4°C using a high-speed centrifuge. The concentration was quantified by bicinchoninic acid (BCA) kit (BCA, Construction, Nanjing, China). First, protein was separated by 10% sodium dodecyl sulfate-polyacrylamide gel electrophoresis (SDS-PAGE). The dispersed proteins were then transferred to a polyvinylidene fluoride membrane for 4 h at 4°C. Incubate with 5% skim milk for 1 h. Second, incubate the membrane with a special primary antibody (PFKM, Abcam, Cambridge, MA, USA, Rabbit, 1 : 3000; SOD1, Abcam, Cambridge, MA, USA, Rabbit, 1 : 3000; SOD2, Abcam, Cambridge, MA, USA, Rabbit, 1 : 3000; AMPK, Abcam, Cambridge, MA, USA, Rabbit, 1 : 3000; and p-AMPK, Abcam, Cambridge, MA, USA, Rabbit, 1 : 3000). We rinse the membrane 3 times with tris buffered saline-tween (TBST) for 15 minutes each time. Next day, the membrane was bound to a second antibody for 1 h at 37°C and 3 times with TBST. Visual inspection of proteins was by the electrochemiluminescence (ECL) Plus detection system.

### 2.10. Statistical Analysis

All statistical results were presented as mean ± SD. The GraphPad Prism 5 Software (La Jolla, CA, USA) was used. Student's *t*-test was used to analyze the comparison between the two groups. *P* values less than 0.05 were considered statistically significant results.

## 3. Results

### 3.1. PFKM Can Be Predicted Involving in Regulating the Oxidative Stress

The miRDB database and the TargetScan database were used to predict the target gene of miR-383-5p. In addition, the AMPK pathway is thought to be associated with the development of AMI. We found that PFKM was located in the AMPK signaling pathway.

### 3.2. PFKM Is a Target Gene of miR-383-5p

**Figure 1:**
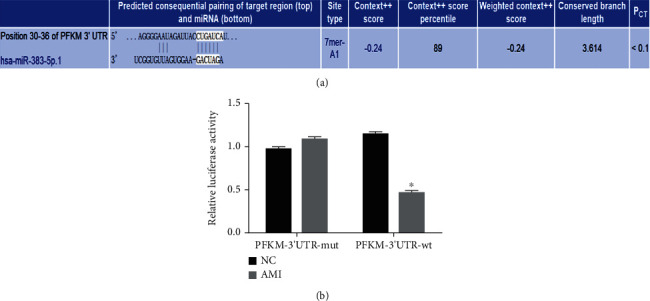
PFKM is a target gene of miR-383-5p. (a) The predicted binding sites of miR-383-5p on the 3′UTR sequence of PFKM gene. (b) The luciferase activity of PFKM-3′UTR-wt and PFKM-3′UTR-mut; ^∗^*P* < 0.05 vs. the NC group; UTR: untranslated region; wt: wild type; mut: mutant type; miR-383-5p: microRNA-383-5p; PFKM: phosphofructokinase muscle-type; NC: negative control. The results of luciferase activity were regarded as measurement data, expressed as mean ± SD and analyzed using the *t*-test, and the experiment was repeated 3 times.

We found that there is a specific binding region between the 3′UTR of PFKM and the miR-383-5p sequence through software analysis. So, we hypothesized that PFKM was a direct target gene of miR-383-5p (Figure 1(a)). We then used the dual luciferase reporter assay, and the results showed that miR-383-5p significantly downregulated PFKM-3′UT-wt luciferase activity compared to NC group (*P* < 0.05), but PFKM-3′UT-mut luciferase activity had no significant effect (*P* > 0.05). Therefore, we can obtain that miR-383-5p can specifically bind to PFKM-3′UTR, thereby inhibiting PFKM gene expression (Figure 1(b)).

### 3.3. H_2_O_2_-Induced Upregulation of miR-383-5p Expression and Increased PFKM Expression in H9C2 Cells

**Figure 2:**
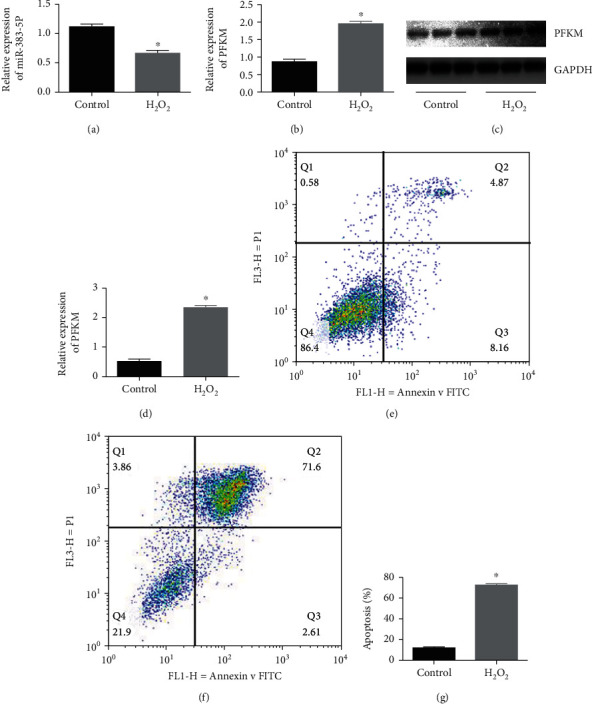
miR-383-5p expresses poorly while PFKM expresses highly in H_2_O_2_-induced H9C2 cells. (a, b) The expression of miR-383-5p and PFKM in normal H9C2 cells and H_2_O_2_-induced H9C2 cells, H9C2 cells detected by RT-qPCR. (c, d) The protein expression of PFKM in normal H9C2 cells and H_2_O_2_-induced H9C2 cells examined by western blot analysis, with band intensity assessed. (e) and (f) The representative images of flow cytometry using Annexin V-FITC and PI staining. (g) Statistical analysis of apoptosis rate detected *via* flow cytometry. ^∗^*P* < 0.05 vs. the control group. The data were expressed as mean ± SD. Data at different time points were compared using repeated measurements ANOVA. The experiment was repeated 3 times. miR-383-5p: microRNA-383-5p; PFKM: phosphofructokinase muscle-type.

Real-time qPCR and western blot were used to detect the expression of miR-383-5p and PFKM in normal H9C2 cells and H_2_O_2_-induced H9C2 cells at 4 h. And results showed that miR-383-5p expression was decreased (*P* < 0.05), but mRNA and protein expression levels of PFKM were significantly elevated in H_2_O_2_-induced H9C2 cells (*P* < 0.05) (Figures 2(a)–2(d)). Also, we did flow cytometry to detect the rate of apoptosis, and the result showed that apoptosis rate significantly increased in the H_2_O_2_ group (Figures 2(e)–2(g)).

### 3.4. miR-383-5p Regulates the Expression of the AMPK Signaling Pathway Factors by Targeting PFKM

**Figure 3:**
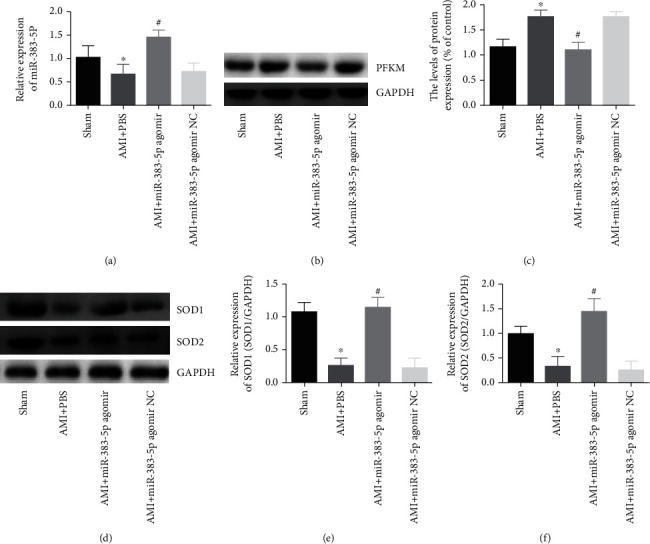
Upregulation of miR-383-5p suppresses PFKM. The myocardial tissues used for following detections were, respectively, treated with sham, AMI+PBS, AMI+miR-383-5p agomir, and AMI+miR-383-5p agomir NC. (a) miR-383-5p expression of myocardial tissue of mice by RT-qPCR. (b, c) Protein expression of PFKM in the myocardial tissue of mice by western blot analysis. (d) SOD1 and SOD2 expression by western blot. (e, f) The mRNA expression results of SOD1 and SOD2 in the three groups were determined by real-time PCR. ^∗^*P* < 0.05 vs. the sham group; ^#^*P* < 0.05 vs. the AMI+PBS group; the results of miR-383-5p expression, PFKM mRNA, and protein expressions were regarded as measurement data, presented by mean ± SD and analyzed by one-way ANOVA. There were 10 mice in each group, and the experiment was repeated 3 times; miR-383-5p: microRNA-383-5p; PFKM: phosphofructokinase muscle-type; AMI: acute myocardial infarction.

We used real-time qPCR to detect the expression of related genes to research the effect of miR-383-5p on AMPK signaling pathway. The results showed (Figure 3(a)) that compared with the sham group, the expression of miR-383-5p was significantly decreased in the AMI+PBS group (*P* < 0.05). In the AMI+miR-383-5p agomir group, miR-383-5p expression was significantly elevated (*P* < 0.05). At the same time, there was no significant difference of miR-383-5p expression in the AMI+miR-383-5p agomir NC group (*P* > 0.05). Western blot (Figures 3(b) and 3(c)) result showed that miR-383-5p agomir inhibited PFKM expression after AMI. In the miR-383-5p agomir group, the expression of SOD1 and SOD2 was increased. However, we found no significant difference in the AMI+miR-383-5p agomir NC group (*P* > 0.05) (Figure 3(d)). mRNA also obtained similar results (Figures 3(e) and 3(f)). We can conclude that miR-383-5p overexpression can inhibit oxidative stress after AMI.

### 3.5. miR-383-5p Reduces the Effects of Oxidative Stress after AMI through the AMPK Signaling Pathway

**Figure 4:**
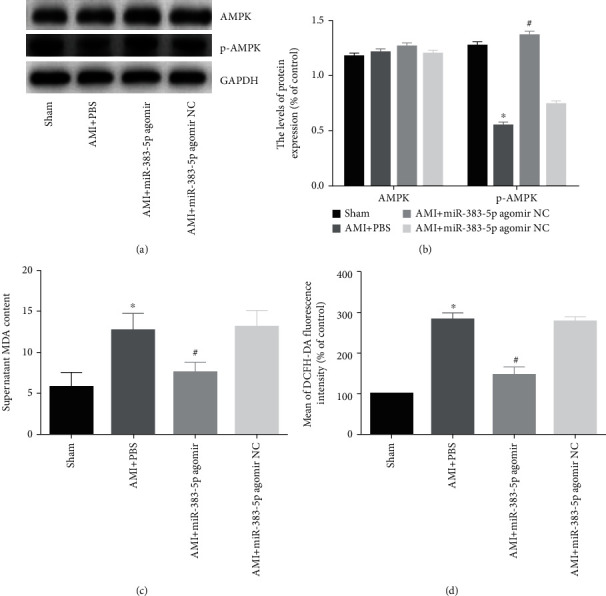
miR-383-5p reduces the effects of oxidative stress through the AMPK signaling pathway. The myocardial tissues used for following detections were, respectively, treated with sham, AMI+PBS, AMI+miR-383-5p agomir, and AMI+miR-383-5p agomir NC. (a, b) Protein expression of AMPK and p-AMPK, in the myocardial tissue of mice by western blot analysis. (c) MDA kit was used to detect the expression level of MDA in the 4 groups. (d) Flow cytometry was used to detect ROS levels in the 4 groups of myocardial tissues. ^∗^*P* < 0.05 vs. the sham group; ^#^*P* > 0.05 vs. the AMI+PBS; AMPK and p-AMPK protein expressions were regarded as measurement data, presented by mean ± SD and analyzed by one-way ANOVA. There were 10 mice in each group, and the experiment was repeated 3 times; miR-383-5p: microRNA-383-5p; AMPK: AMP-activated protein kinase; p-AMPK: phosphorylated AMP-activated protein kinase; MDA: malondialdehyde; ROS: reactive oxygen species.

Moreover, the phosphorylation of AMPK was significantly increased in the AMI+miR-383-5p agomir group (*P* < 0.05). In conclusion, miR-383-5p overexpression inhibits PFKM expression after AMI and activates AMPK signaling pathway, which in turn affects downstream factors (*P* < 0.05) (Figures 4(a) and 4(b)). We all know that the level of MDA and ROS can reflect the oxidative stress level; then, we used MDA kit to study the effect of miR-383-5p on MDA and used flow cytometry to determine the level of ROS. The results (Figure 4(c) and 4(d)) showed that MDA and ROS levels increased in the AMI+PBS group (*P* < 0.05), and by contrast, MDA and ROS levels decreased significantly in the AMI+miR-383-5p agomir group (*P* < 0.05). miR-383-5p could decrease the levels of MDA and ROS through AMPK signaling pathway, thereby reducing oxidative stress of myocardium.

### 3.6. Overregulation of miR-383-5p Increased Cardiac Function in Mice

**Figure 5:**
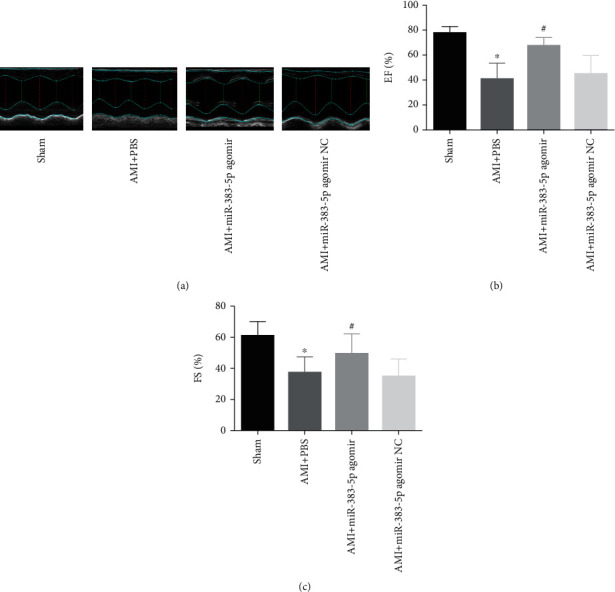
Overexpression of miR-383-p increases the cardiac function in mice. The myocardial tissues used for following detections were, respectively, treated with sham, AMI+PBS, AMI+miR-383-5p agomir, and AMI+miR-383-5p agomir NC. (a) Representative photographs in mouse echocardiography. (b) Cardiac function index EF of mice. (c) Cardiac function index FS of mice. There were 10 mice in each group, and the experiment was repeated 3 times; miR-383-5p: microRNA-383-5p; EF: ejection fraction; FS: fraction shortening.

We used echocardiography to examine the heart function of mice (Figure 5(a)). The results confirmed that the AMI+miR-383-5p agomir group had a significant increase in cardiac function (Figures 5(b) and 5(c)): EF and FS (*P* > 0.05). In the AMI+miR-383-5p agomir NC group, the results had no significant difference (*P* > 0.05). And this study indicated that miR-383-5p agomir can relieve cardiac function after AMI.

## 4. Discussion

In this study, we demonstrated that miR-383-5p could reduce the oxidative stress after AMI through AMPK signaling pathway by targeting PFKM, thereby alleviating the redox imbalance produced by the heart after AMI and inhibiting cardiomyocyte apoptosis.

Our study demonstrated that increasing the expression of miR-383-5p may be a novel approach to AMI treatment strategies. Previous researches have found that abnormal expression of miRs was considered to play an important role in the diagnosis and treatment of various diseases such as heart disease [16–18]. miR-383-5p was known to suppress carcinoma cell proliferation [19, 20] and could be acted as new prognostic biomarkers and therapeutic targets [21]. Furthermore, we used ROS level detection flow cytometry and immunofluorescence to detect intracellular ROS levels, and we used MDA kit to detect the effect of miR-383-5p on MDA levels. Our data suggest that miR-383-5p plays a key role in inhibiting oxidative stress in cardiomyocytes. Secondly, we successfully predicted that PFKM might be the target gene of miR-383-5p through software analysis. Therefore, we conducted a series of studies through *in vivo* and *in vitro* experiments. A lot of studies have investigated that AMPK pathway is involved in the oxidative stress progression [22–24]. And PFKM was the gene enriched in the AMPK signaling pathway by KEGG in AMI. Our study showed that miR-383-5p was decreased after AMI, and when we increased its expression in mouse's heart, cardiac function will be improved. However, decreasing the expression of miR-383-5p after AMI, we did not find the cardiac function deteriorated. And it might be explained by the fact that miR-383-5p has the low expression in the heart. We observed that microRNA-383-5p overexpression alleviates heart injury in vivo. On the one hand, microRNA-383-5p reduced the level of oxidative stress and thus reduces cell apoptosis [25, 26]. In addition, microRNA-383-5p may also reduce cell apoptosis directly.

However, the research also had some limitations. First, we did not have many researches *in vitro*. Second, to detect the level of oxidative stress, we only detect the levels of MDA and ROS, and we did not do other researches in apoptosis such as TUNNEL staining *in vivo*. Finally, this study was still on the way, and the mechanism was insufficient. It needs further verification on the effects of miR-383-5p or AMPK signaling pathway on oxidative stress for the treatment of AMI.

## 5. Conclusions

In conclusion, our study confirmed that miR-383-5p could reduce the oxidative stress after AMI through AMPK signaling pathway by targeting PFKM.

## Figures and Tables

**Table 1:** Real-time PCR primers.

Gene name	Forward (5′>3′)	Reverse (5′>3′)
SOD1	GGTGAACCAGTTGTGTTGTC	CCGTCCTTTCCAGCAGTC
SOD2	CAGACCTGCCTTACGACTATGG	CTCGGTGGCGTTGAGATTGTT
PFKM	CATCGCCGTGTTGACCTCT	CCCGTGAAGATACCAACTCGG
miR-383-5p	GGGAGATCAGAAGGTGATTGTGGCT	CAGTGCGTGTCGTGGAGT
GAPDH	ACAACTTTGGTATCGTGGAAGG	GCCATCACGCCACAGTTTC

RT-PCR: quantitative reverse-transcription polymerase chain reaction.

## Data Availability

The datasets used and analyzed during the current study are available from the corresponding author on reasonable request.
